# The Protective Effect of Grape-Seed Proanthocyanidin Extract on Oxidative Damage Induced by Zearalenone in Kunming Mice Liver

**DOI:** 10.3390/ijms17060808

**Published:** 2016-05-25

**Authors:** Miao Long, Shu-Hua Yang, Jian-Xin Han, Peng Li, Yi Zhang, Shuang Dong, Xinliang Chen, Jiayi Guo, Jun Wang, Jian-Bin He

**Affiliations:** 1 Key Laboratory of Zoonosis of Liaoning Province, College of Animal Science & Veterinary Medicine, Shenyang Agricultural University, Shenyang 110866, China; longjlau@126.com (M.L.); yangshuhua0001@126.com (S.-H.Y.); myworld_a4@126.com (J.-X.H.); lipeng79625@163.com (P.L.); sihuo12345@sohu.com (Y.Z.); DongS_FOCUS@163.com (S.D.); xinliang@syau.edu.cn (X.C.); guoniuniu163@163.com (J.G.); 2 College of Animal Science and Technology, Jilin Agricultural University, Changchun 130118, China

**Keywords:** grape seed proanthocyanidin extract, zearalenone, oxidative damage, Nrf2/ARE pathway, liver, mice

## Abstract

Although grape-seed proanthocyanidin extract (GSPE) demonstrates strong anti-oxidant activity, little research has been done to clearly reveal the protective effects on the hepatotoxicity caused by zearalenone (ZEN). This study is to explore the protective effect of GSPE on ZEN-induced oxidative damage of liver in Kunming mice and the possible protective molecular mechanism of GSPE. The results indicated that GSPE could greatly reduce the ZEN-induced increase of serum aspartate aminotransferase (AST) and alanine aminotransferase (ALT) activities. GSPE also significantly decreased the content of MDA but enhanced the activities of antioxidant enzymes SOD and GSH-Px. The analysis indicated that ZEN decreased both mRNA expression levels and protein expression levels of nuclear erythroid2-related factor2 (Nrf2). Nrf2 is considered to be an essential antioxidative transcription factor, as downstream GSH-Px, γ-glutamyl cysteine synthetase (γ-GCS), hemeoxygenase-1 (HO-1), and quinone oxidoreductase 1 (NQO1) decreased simultaneously, whereas the pre-administration of GSPE groups was shown to elevate these expressions. The results indicated that GSPE exerted a protective effect on ZEN-induced hepatic injury and the mechanism might be related to the activation of the Nrf2/ARE signaling pathway.

## 1. Introduction

The worldwide contamination of grains designated to human and animal feeding with Fusarium mycotoxins is a significant problem. Among Fusarium mycotoxins, zearalenone (ZEN) is the most prevalent mycotoxins found in cereals [1]. ZEN can cause several reproductive disorders in animals by disrupting the endocrine function via binding the estrogen receptors [2]. In addition, many studies have shown that ZEN has hepatotoxic for the liver, which is a primary target [3,4]. ZEN can induce liver lesions and alter part of enzymatic parameters of hepatic function [5,6,7]. Since ZEN can induce lipid peroxidation [8,9], some researchers believe that the oxidative stress may be a key mechanism of toxicity of ZEN *in vivo* and *in vitro* [10,11,12].

In recent years, natural products have been used to improve the health and to prevent diseases, especially those caused by oxidative stress. In this regard, many researchers have reported that the addition of nutrients especially some antioxidants such as Lycopene [13], allium sativum [14], and crocin [15] is one approach that reduces ZEN toxicity.

The natural antioxidant proanthocyanidin contains a variety of phenolic compounds and is often present in vegetables, seeds, tea, fruits, wine, and nuts [16]. Grape-seed proanthocyanidin extract (GSPE), an extract obtained from red grape seeds, which are rich in plant flavonoids, proanthocyanidin oligomers and polymerized oligomers, has been widely marketed in China, [17,18]. GSPE exhibits a wide range of biologic properties for resisting oxidative stress. They can clean off the free radicals and reduce the membrane lipid peroxidation [19,20]. Some research has revealed that GSPE can prevent drug-induced liver and kidney injury and can induce anti-tumor, anti-radiation activity [21,22]. Studies have shown that GSPE can use potent antioxidant features to improve the abnormalities and antioxidant system status of diabetic rats arising from streptozotocin [23], can protect against tissue damage induced by ischemia and/or ischemia/reperfusion in rat ovaries [24], and plays an important role as a potential candidate for the therapy of the metabolic syndrome, such as hypertension and hyperlipidemia [25]. Some research has proven that GSPE is able to conduct PKC and NF-κB inhibition to prevent endothelia dysfunction induced by high glucose [26] and utilize the activation of the Nrf2 pathway to improve diabetic bladder dysfunction [27]. However, there are few reports about GSPE’s ability to alleviate the toxicity of mycotoxins. Although our previous study showed that GSPE could protect Aflatoxin B1-induced subchronic immune injury of mice [28], the protective effects of GSPE on ZEN *in vivo* induced hepatotoxicity, and its role of mechanism have rarely been studied.

It is known that liver injury induced by ZEN is mainly led by oxidative stress. As current existing research results indicate, Nrf2 is a transcription factor of adjusting redox status and the cellular antioxidative responses via increasing the antioxidative genes expression by using antioxidant response element (ARE) [29,30]. To date, it has been shown that ZEN can induce oxidative damage involving the Nrf2/ARE pathway, as has been demonstrated in cell culture systems [31], and that ZEN mediated toxicity found in the jejunum is generated due to one of the mechanism-signal pathways mediated by Nrf2 [32].

It is assumed that ZEN-induced toxicity in liver can be protected by GSPE through counteracting oxidative injury by regulating the Nrf2 pathway.

Therefore, this research explored the role that ZEN plays in the oxidative status of liver and investigated the Nrf2 signal pathway in mice. Moreover, the authors concluded that the activation of the Nrf2 signal pathway possibly leads to the protective effect of GSPE. The results can provide the protective effect of mechanisms of GSPE on ZEN-induced liver injury mice.

## 2. Results

### 2.1. Effects of Aspartate Aminotransferase (AST) and Alanine Aminotransferase (ALT) on the Serum Activities

Serum activities of aspartate aminotransferase (AST) and alanine aminotransferase (ALT) function as the biochemical signs for revealing hepatic damage. In comparison with the other groups (*p* < 0.05), both ALT and AST in the ZEN group had shown a greatly increased serum activity (Table 1). However, by carrying out pre-administration of GSPE for five days, the increase in the serum activity of both ALT and AST can be significantly prevented due to the effects of ZEN. In the control group, administration of GSPE (100 mg/kg) had little effect on the alteration of the level of hepatic markers (*p* > 0.05).

### 2.2. The Effect on Antioxidant of Liver

The hepatic level of liver malondialdehyde (MDA) is used as an index indicating the lipid peroxidation in liver damage. Compared with the control group (*p* < 0.05), the administration of ZEN gave rise to an apparent rise of the MDA content in liver, while the pre-administration of GSPE greatly reduced the MDA levels (Table 2). In addition, compared with the control group (*p* < 0.05), the hepatic activities of both superoxide dismutase (SOD) and glutathione peroxidase (GSH-Px) declined after ZEN treatment, and the pre-administration of GSPE remarkably reversed the reduced activities of GSH-Px and SOD (*p* < 0.05) (Table 2). The findings obtained confirmed the effects of GSPE in significant improvement of the antioxidant activities in the liver, and the decrease in the serious ZEN induced oxidative damage.

### 2.3. Effect on Histopathological Variation in Liver

**Figure 1:**
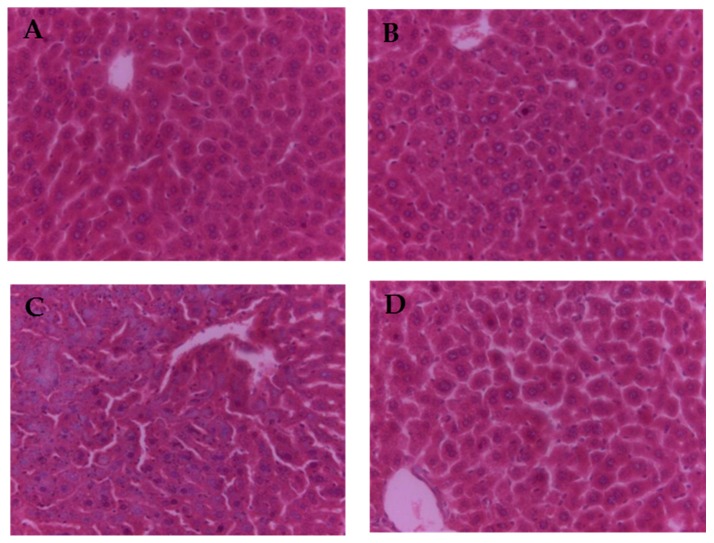
Pretreatment effects of GSPE on ZEN-induced liver histopathological changes in mice (original magnification of ×400). (**A**) control group; (**B**) GSPE group; (**C**) group administrated with ZEN at a dose of 40 mg/kg; (**D**) pre-administrated with GSPE at a dose of 100 mg/kg treatment group.

The results of liver sections with regard to the histopathological aspect were shown in Figure 1. The morphology of liver in control group and GSPE group exhibited normal hepatic cells with a clear cell nucleus and well-preserved cytoplasm (Figure 1A,B). In contrast, the ZEN group revealed the damage of liver tissues; the liver tissue displayed the disorder of the hepatic cell cords, the central venous border was not clear, the chromatin was lightly stained and dissolved, and the nuclear membrane disappeared (Figure 1C). However, the protection was observed in the pre-administration of GSPE; the liver sections of the mice from this group showed minor pathomorphological changes (Figure 1D).

### 2.4. Effect on the Gene Expression Associated with the Nrf2 Signaling Pathway

**Figure 2:**
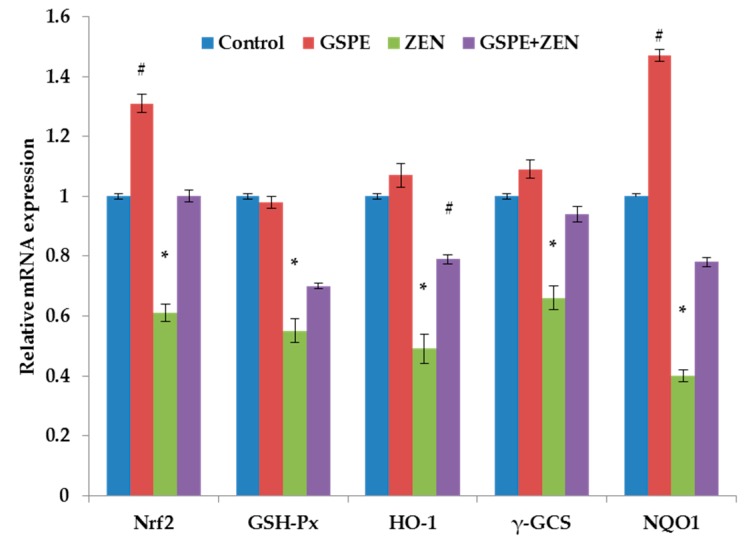
Pretreatment effects of GSPE on ZEN-induced the relative mRNA expression of the nuclear erythroid2-related factor2 (Nrf2), glutathione peroxidase (GSH-Px), hemeoxygenase-1 (HO-1), γ-glutamyl cysteine synthetase (γ-GCS), and quinone oxidoreductase 1 (NQO1) in the liver of mice. Values are mean ± SEM of ten mice in each group. * *p* < 0.05 *vs.* control group, ^#^ *p* < 0.05 *vs.* ZEN-treated group.

The role of GSPE that plays in genes associated with the Nrf2 signaling pathway is illustrated in Figure 1. In comparison with the control group (*p* < 0.05), expression levels of Nrf2, GSH-Px, HO-1, γ-GCS, and NQO1 in the ZEN group were remarkably lower; in contrast, the usage of GSPE greatly rose the expression levels of Nrf2, GSH-Px, HO-1, γ-GCS, and NQO1. Pre-administration of GSPE groups showed a greater increase in the genes expression correlated to the Nrf2 signaling pathway than that of the ZEN group (*p* < 0.05; Figure 2).

### 2.5. Effect on Nrf2 Signaling-Related Protein Expressions

**Figure 3:**
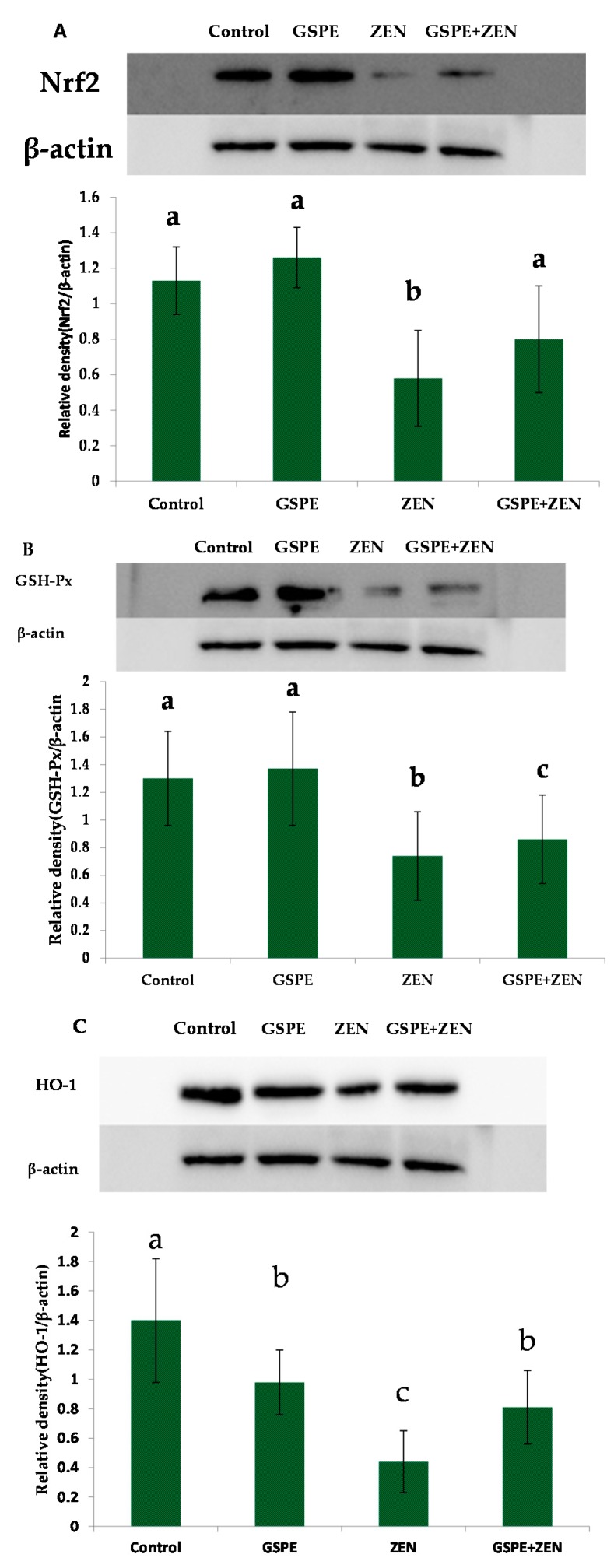

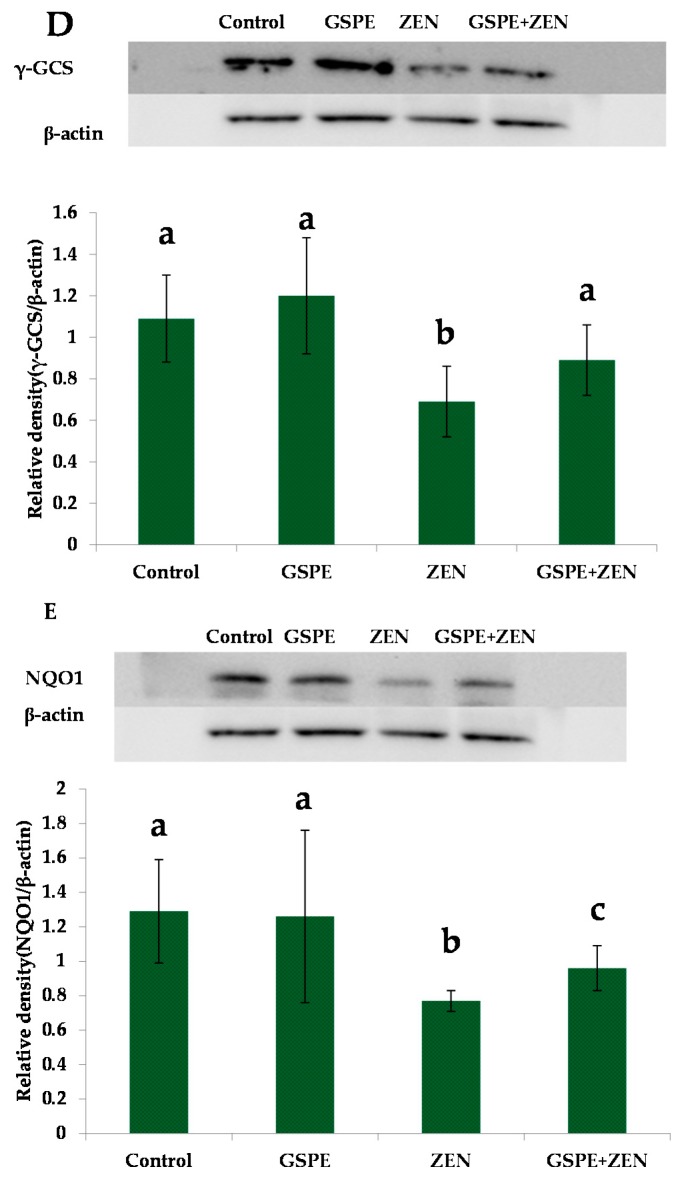
Pretreatment effects of GSPE on ZEN-induced the protein expression levels of the Nrf2/ARE signaling pathway in the liver of mice. (**A**) Nuclear erythroid2-related factor2 (Nrf2); (**B**) glutathione peroxidase (GSH-Px); (**C**) hemeoxygenase-1 (HO-1); (**D**) γ-glutamyl cysteine synthetase (γ-GCS) and (**E**) quinone oxidoreductase 1 (NQO1). Values expressed as mean ± SE in each group. Values are mean ± SEM of ten mice in each group. ^a,b,c^ Means with different letters are significantly different, *p* < 0.05.

To analyze whether Nrf2 activation plays a role in GSPE protection against the toxicity caused by ZEN, we measured the expression of Nrf2 and the Nrf2-target proteins—GSH-Px, HO-1, NQO1, and γ-GCS in mice livers. Western bolt study revealed that ZEN exerted an inhibitory effect on Nrf2 protein expression and also its down-stream target proteins GSH-Px, HO-1, γ-GCS, and NQO1. As can be seen in Figure 3A, the treatment with ZEN could greatly down-regulated the protein levels of hepatic Nrf2 in comparison with the control group elevated by the use of GSPE. Compared with the control group, the expressions of protein GSH-Px, HO-1, γ-GCS, and NQO1 showed a significant reduction in the ZEN group, as seen from Figure 3B–E. Although pre-administration of GSPE did not significantly reverse the decreased expression (Figure 3B–E), these protein expressions were higher than that in ZEN group.

## 3. Discussion

In this work, the doses of ZEN (40 mg/kg-8% of LD_50_) were chosen based on preliminary experiments by Boeira *et al.* [33]. However, the dose gradients were not employed in the GSPE administration. This is because our previous studies had found that GSPE exhibited an excellent protective effect and was shown to be safe at the dose of 100 mg/kg [28]; in this present study, it was shown that oral administration of a dose of 100 mg/kg GSPE to mice also had better protective effects on liver damage.

One study indicates that ZEN shows hepato-nephrotoxicity and can affect the enzymatic and hematological parameters of mice within 48 h in the case of oral administration [34]. In the present work, oral administration of ZEN was found to greatly improve serum activities of both ALT and AST at the end of our experiment. AST and ALT, as markers for revealing the liver health, can be observed in both cytoplasm and mitochondria of hepatocytes. Thus, the elevation of the two enzymes activities in the serum after 48-h treatment with ZEN indicated that ZEN could change the organ pathology and induce the damage to livers. The findings were consistent with the report [34]. Pre-administration of GSPE can largely reduce the serum activities caused by ZEN in ALT and AST. The results revealed that GSPE plays a protective role in the resistance to the liver injury induced by ZEN. Furthermore, this is the first time it has been reported that GSPE can protective the liver injury of mice induced by ZEN. However, in our study, the liver of mice showed a slight histopathological variation for a short period. This may be related to the dose and counteracting time of ZEN. In general, histopathological results for the liver sections confirmed the protective effect of GSPE on the hepatic injury caused by ZEN (Figure 1D).

One study reported that ZEN induced oxidative stress mainly contributes to liver damage [10]. The parameters of oxidative stress, including GSH-Px, SOD, and MDA, are often used to measure the oxidative injury of organs especially the liver. MDA is often seen as a biomarker for oxidative stress and a key feature in liver injury, as it is an end of lipid peroxidation product and is also regarded as the cellular pathways contributing to oxidative [35]. Similar to the existing literature, the findings in our research indicated that the MDA levels in the liver were higher in the ZEN group than those in the control group [4,12]. Our results showed that pre-treated GSPE largely restrained the elevation of MDA in the mice livers in the ZEN-treated group. SOD can represent an important antioxidant defense in almost all cells that are exposed to oxygen, and GSH-Px, as a enzyme, can get rid of the excessive H_2_O_2_ generated from the disputation of O^2−^ following the administration of MDA. In the present work, the analysis verified that SOD and GSH-Px activities showed significant reduction in the liver treated with ZEN. The results indicated that ZEN made the liver subject to the oxidative damage. The results were consistent with previous studies [4,7,10]. In the present study, GSPE largely promoted the activities of SOD and GSH-Px in the mice livers. Our results suggested that GSPE has a potent ability to ameliorate oxidative stress-induced liver damage through reducing lipid peroxide and enhancing the antioxidative enzymes activities in mice livers.

The Nrf2/ARE pathways have an anti-oxidative effect on alleviating toxicant-induced hepatotoxicity [36]. The activation in the Nrf2 signaling pathway was able to prevent the cells from oxidative stress damage. Some researchers have proposed that Nrf2 can activate the antioxidative stress system to excrete the toxic metabolites via regulating the expression of many intracellular antioxidant genes [37]. The genes of GSH-Px, HO-1, γ-GCS, and NQO1, belonging to the ARE, are the downstream target genes of Nrf2. These proteins have been known to be the cytoprotective effect resistant to oxidative stress [38,39].

The induction of these enzymes can be mainly realized by the bind of Nrf2 and the ARE present in the upstream regulatory region for many phase 2 genes. Our results showed that GSPE improved the decreased downstream target genes (phase 2 genes) mRNA levels of Nrf2 including GSH-Px, HO-1, γ-GCS, and NQO1 caused by ZEN (Figure 2). It indicated that GSPE could activate the Nrf2 expression because these phase 2 genes are shown to be Nrf2-dependent. Based on the upstream regulatory region, mRNA levels of phase 2 genes are regulated, and the ARE is controlled by Nrf2.

In the literature, it has been proposed that the declined nuclear Nrf2 may be led by the decrease in the nuclear export and Nrf2 mRNA or in Nrf2 proteins [38]. Our results showed that GSPE improved both the levels for Nrf2 mRNA and the Nrf2 protein expression decreased by ZEN. These results indicated that elevating the accumulation of proteins of Nrf2 in the nucleus might be through increasing the expression levels of Nrf2 by GSPE. Kwak *et al.* (2002) [40] reported that Nrf2 regulates its expressions by itself using an ARE-like elements present in the proximal region of its promoters. This makes Nrf2 have persistent nuclear accumulation and extend the induction for the phase 2 genes regarding toxicity. This viewpoint can explain why the levels of mRNA and the protein of Nrf2 were both increased in our results. However, through which pathway GSPE activated the Nrf2 needs to be researched. Meanwhile, in a future study, we should also study whether GSPE could inhibit Nrf2 ubiquitination and degradation, which might explain the regulative role of GSPE that results in the Nrf2 proteins accumulated in these cells.

It is known that the toxicant-induced liver injuries of animals can be prevented by activation of the Nrf2/ARE signaling pathways. However, the possibility and mechanism of ZEN-induced hepatotoxicity led by Nrf2/ARE signaling pathways is unknown. The results showed that ZEN caused oxidative damage of the liver as indicated by ALT, GSH-Px, MDA levels, AST, and SOD. Moreover, this research indicated that ZEN plays its toxic roles through inhabiting the mRNA expression and the protein levels of Nrf2, GSH-Px, HO-1, γ-GCS, and NQO1. However, Liu *et al.* (2014) reported that the Nrf2 expression both in mRNA and protein levels were increased in intestinal tissue of rats which were induced by ZEN [32]. The differences of the two results might be that the doses of ZEN and the challenge time were different. Although these results were different, they proved that the oxidative toxicity of ZEN was related to the Nrf2/ARE. Moreover, the results of the current study showed that GSPE was an Nrf2 activator and the activation had an essential effect on the hepatoprotection mediated by GSPE against the liver oxidative damage caused by ZEN. Hence, the findings revealed that GSPE has a hepato-protective effect on the toxicity induced by ZEN, which may have resulted from the NRF2/ARE pathways.

## 4. Experimental Section

### 4.1. Animals

We purchased the male Kunming mice (45 ± 2 g and 8 weeks-old) from the Experimental Animal Center of China Medical University, Shenyang, China. At firstly, the mice were bred in a room which is restricted-access, for 12-h light/dark cycles with a humidity of 40%–60% at a temperature ranging from 22 to 24 °C. By providing water and diet on the condition of minimum *ad labitum* and all stress factors, the acclimatization was conducted on the mice for 1 week prior to the experiments. These experiments were performed subject to the European Communities Council Directive of 24 November 1986 (86/609/EEC) and the principles of good laboratory animal care. Moreover, the experiments were permitted by the ethics committee for laboratory animal care for the use of Shenyang Agricultural University, Shenyang, China.

### 4.2. Chemicals

In this work, based on the ZEN obtained from Sigma (St. Louis, MO, USA), we prepared the stock solution of 200 mg/mL ZEN in diethyl sulfoxide, and the solution was stored at −20 °C. By dispensing the stock solution into the sterilized peanut oil, we obtained the working solution. GSPE with a purity equal or greater than 95% was purchased from Nanjing Zelang Medical Technology Co., Ltd., Nanjing, China. The kits used for measuring GSH-Px, SOD, and MDA activities were obtained from the Nanjing Jiancheng Bioengineering Institute (Nanjing, China); SYBR green RT-PCR kit from Takara (Otsu, Japan) and DAPI from Sigma Aldrich (St. Louis, MO, USA) were also employed. The primers for Nrf2, GSH-Px, HO-1, γ-GCS, NQO1, and β-actin were synthesized and purified by Sangon Biotech (Shanghai, China); moreover, the preservation solution of RNA samples and the kits for total animal RNA extraction were obtained from Sangon Biotech (Shanghai) Co., Ltd, Shanghai, China. The Kits for Revert Aid First Strand cDNA Synthesis were purchased from MBI Fermentas (Burlington, ON, Canada); the mice anti-Nrf2, γ-GCS, GSH-Px antibodies were acquired from Santa cruz biotechnology (Santa, Dallas, TX, USA). The catatory number of the anti-Nrf2 antibody was sc-722, the anti-γ-GCS antibody was sc-22755, and the anti-GSH-Px antibody was sc-30147. Anti- HO-1, NQO1, and β-actin antibodies were from Sangon Biotech (Shanghai, China); these antibodies were all polyclonal antibodies. We also purchased the antibodies conjugated with the secondary goat anti-mouse and goat anti-rabbit horseradish peroxidase (HRP) in Beijing Solarbio Science & Technology Co., Ltd. (Beijing, China).

### 4.3. Experimental Design and Treatment

The control group (*n* = 10) was orally administrated with physiological saline every day for 7 days.

The ZEN group (*n* = 10) was orally administrated with physiological saline every day for 5 days and, with a 40 mg/kg dose of ZEN (40 mg/kg-8% of LD_50_), was based on preliminary experiments by Boeira *et al.* (2014, 2015) [13,33] on the 6th day and 7th day.

The GSPE (*n* = 10) was orally administrated with a 100 mg/kg dose of intragastric GSPE diluted with physiological saline daily, for 5 days, and then orally administrated with physiological saline for 2 days.

The GSPE+ZEN group (*n* = 10) was orally administrated with a 40-mg/kg dose of ZEN on the 6th day and 7th day and with a 100-mg/kg dose of intragastric GSPE diluted with physiological saline daily for 5 days.

The mice were found to die subject to the ether anesthesia after the administration for 48 h. Afterwards, we collected the blood samples and soon separated the serum. Lastly, the liver tissues were stored at −80 °C for further use in the experiments after being isolated from each mouse.

### 4.4. Parameters

In the experiment, we evaluated the oxidant levels of the mice liver based on the content of the MDA and examined the enzyme levels of antioxidants in the liver by analyzing the activities of GSH-Px and SOD. SOD, MDA, and GSH-Px assay kits were used to carry out the analysis. By investigating the AST and ALT levels, we assessed the liver function. The relevant diagnostic kits were adopted in the analysis of these indexes. The details of all determination procedures followed the manufacturer’s instructions for the commercial kits.

### 4.5. Histopathology Examination of Liver Tissue

Before conducting routine processing and paraffin embedding, the liver section was set in 10% formalin. We then used hematoxylin and eosin to stain the liver sections and investigated them by using a photomicroscope.

### 4.6. Gene Expression

TRIzol reagent was used to extract the total RNA of the livers. Then, the purity of the total RNA was measured via the quotient for OD at 260/280 nm. Moreover, the TaKaRa PrimeScript RT reagent kit was adopted to perform reverse transcription. The mRNA contents of β-actin, HO-1, GSH-Px, NQO1, Nrf2, and γ-GCS in the mice livers had been measured using a quantitative real-time PCR. To normalize the data of the gene expression, β-actin was employed as a housekeeping gene. Based on the PubMed database, we acquired the primer information regarding all the genes. The application software primer 5 was used to design the primers, and the Oligo 7 was used to test the specificity of the primers (Table 3). An ABI 7500 real-time PCR system and the SYBR Green PCR Kit were used to conduct real-time PCR. Each sample had been measured in triplicate. Afterwards, a method of gene expression (*i.e.*, 2^−∆∆*C*t^) was utilized to analyze the data of real-time PCR. The results revealed that there was a fold change shown in the expression of genes which were normalized to the endogenous reference genes (β-actin) and function as a calibrator. The system of the reaction mixtures consisted of: 2 μL product of cDNA, 0.4 μL reverse primers, 0.4 μL forward, 10 μL Taq MasterMix solution, 6.8 μL of RNase-free water, and 0.4 μL Rox. The conditions of conducting the PCR reaction included: at the initial stage, denaturating at 95 °C for 5 min, and then denaturating at 95 °C for 10 s, annealing at 95 °C for 5 s, and extension at 60 °C for 34 s. The amount of the template was measured based on the standard curve of quantitative analysis.

### 4.7. Western Blot Assay

We harvested all protein extracts of the liver tissues and carried out homogenization in RIPA lysis buffer. The nuclear/cytoplasmic proteins in the liver tissues were obtained by the protein extraction kit after centrifuging the 12,000× g lysates at 4 °C for 10 min. The extraction procedure was performed strictly based on the instructions of manufacturers. The BCA protein assay kit was used to determine the protein concentrations. The proteins of the liver tissue were separated by SDS-PAGE. After this procedure, the proteins were transferred to PVDF membranes. The blocking solution contained with 5% skimmed milk was used to block the membranes for 1 h at room temperature. Afterwards, we incubated the membranes overnight at 4 °C using primary antibodies for Nrf2, GSH-Px, HO-1, γ-GCS, NQO1, and β-actin in blocking solution. Then, the membranes were incubated with secondary antibody blocking solution for 1 h at room temperature. The improved Western blot kits of chemiluminescence detection were used to visualize the proteins. The Image-Analysis system was used to quantify the corresponding expression of target proteins.

### 4.8. Statistical Analysis

Results were presented as the mean ± standard error (X ± SE). Firstly, the authors used one-way ANOVA to assess the significance of differences among mean values. Afterwards, multiple pair-wise comparisons were made using a Student-Newman-Keuls (SNK) *post-hoc* test or the least significant difference (LSD). In addition, SPSS 13.0 software was used to carry out all statistical tests. Mean values were proven to be significantly different at *p* < 0.05.

## 5. Conclusions

In summary, this is the first study to evaluate the antioxidant activity of GSPE against ZEN-induced liver toxicity in mice. Moreover, its hepatoprotective effect might be attributed to the Nrf2/ARE signaling pathways activated.

## Figures and Tables

**Table 1:** Effect of grape-seed proanthocyanidin extract (GSPE) on zearalenone (ZEN)-induced hepatic damage by testing the serum activities of ALT and AST in mice.

Group	ALT	AST
Control	210.5 ± 12.28 ^a^	89.27 ± 7.20 ^a^
GSPE (100 mg/kg)	204.71 ± 10.12 ^a^	95.38 ± 16.68 ^a^
ZEN (40 mg/kg)	288.65 ± 34.57 ^b^	114.19 ± 15.64 ^b^
GSPE (100 mg/kg) + ZEN (40 mg/kg)	235.60 ± 21.25 ^c^	97.56 ± 12.13 ^a^

^a,b,c^ Means within the column with different letters are significantly different, *p* < 0.05. AST: aspartate aminotransferase; ALT: alanine aminotransferase.

**Table 2:** Effect of GSPE on liver antioxidant parameters in mice induced by ZEN.

Group	MDA (nmol/mgprot)	SOD (U/mgprot)	GSH-Px (U/mgprot)
Control	7.03 ± 1.29 ^a^	36.32 ± 3.32 ^a^	213.65 ± 10.12 ^a^
GSPE (100 mg/kg)	6.21 ± 0.57 ^a^	40.26 ± 2.21 ^b^	236.15 ± 22.22 ^b^
ZEN (40 mg/kg)	9.81 ± 1.55 ^b^	32.58 ± 3.11 ^c^	193.64 ± 8.63 ^c^
GSPE (100 mg/kg) + ZEN (40 mg/kg)	8.07 ± 0.89 ^a^	33.99 ± 0.99 ^a^	207.95 ± 8.59 ^a^

^a,b,c^ Means within the column with different letters are significantly different, *p* < 0.05. MDA: malondialdehyde; GSH-Px: glutathione peroxidase; SOD: superoxide dismutase.

**Table 3:** Primers for real-time PCR analyses.

Gene	Accession No.	Primer Sequences (5′-3′)	Product Size/bp
Nrf2	NM_010902.3	F: TCCTATGCGTGAATCCCAAT	103 bp
		R: GCGGCTTGAATGTTTGTCTT	
GSH-Px	X03920.1	F: GAAGTGCGAAGTGAATGG	224 bp
		R: TGTCGATGGTACGAAAGC	
HO-1	NM_010442.2	F: GGGCTGTGAACTCTGTCCAAT	162 bp
		R: GGTGAGGGAACTGTGTCAGG	
γ-GCS	U85414.1	F: TGGATGATGCCAACGAGTC	185 bp
		R: CCTAGTGAGCAGTACCACGAATA	
NQO1	NM_008706.5	F: TTCTGTGGCTTCCAGGTCTT	104 bp
		R: TCCAGACGTTTCTTCCATCC	
β-actin	BC138614.1	F: CTGTCCCTGTATGCCTCTG	221 bp
		R: TTGATGTCACGCACGATT	
